# Prevalence and Factors Associated With Alcohol Use Disorder Among People Attending Primary Health Care Facilities in Rupandehi District, Nepal

**DOI:** 10.1155/ghe3/2790450

**Published:** 2025-09-30

**Authors:** Chet Kant Bhusal, Sigma Bhattarai, Savyata Panthi, Ashok Chhatkuli, Aishwarya Verma, Ananya Kunwar Chhetri, Anwiti Parajuli, Ayushma Khanal, Jainab Khan, Madhav Basyal, Sagar Panta

**Affiliations:** ^1^Department of Community Medicine, Universal College of Medical Science and Teaching Hospital, Tribhuvan University, Bhairahawa, Rupandehi, Nepal; ^2^Department of Nursing, Universal College of Medical Science and Teaching Hospital, Tribhuvan University, Bhairahawa, Rupandehi, Nepal; ^3^Ministry of Health, Koshi Province, Biratnagar, Nepal; ^4^Universal College of Medical Science and Teaching Hospital, Tribhuvan University, Bhairahawa, Rupandehi, Nepal

**Keywords:** alcohol use disorder, hazardous drinking, Nepal, primary healthcare, screening

## Abstract

**Background:**

Alcohol use disorder (AUD) is a global public health issue, impacting individuals physiologically, socially, and mentally. Limited studies were conducted to explore associated factors in Nepal. This study aims to assess AUD prevalence and associated factors among people attending primary healthcare services in Rupandehi, Nepal.

**Methods:**

An institutional-based cross-sectional study was conducted among 688 individuals attending primary healthcare facilities of Rupandehi district, Nepal, using multistage probability sampling technique. The study utilized validated AUD identification test (AUDIT) in its Nepali version for screening AUD. To assess relationships between dependent and independent variables, bivariate analysis was initially conducted. Variables that showed significant association with dependent variable having *p* value < 0.05 were then included in a multivariate logistic regression model to identify final associated factors.

**Results:**

The prevalence of AUD was 30.8% (CI: 27.4–34.4). About 62.8% are in low risk, 26.7% higher risk, 5.1% harmful and hazardous, and 5.4% in alcohol dependence. Respondents aged ≥ 50 years (adjusted odds ratio [AOR] = 0.26, CI: 0.11–0.61), female (AOR = 0.14, CI: 0.07–0.28), non-Hindu (AOR = 0.05, CI: 0.01–0.43), ≥ SLC education (AOR = 0.16, CI: 0.08–0.31) were negatively associated with AUD. Whereas, Newar (AOR = 4.10, CI: 1.00–16.88), rural areas (AOR = 1.57, CI: 1.02–2.42), joint family (AOR = 1.58, CI: 1.05–2.37), daily wages (AOR = 3.57, CI: 1.10–11.56), food sufficiency of 6–9 months (AOR = 1.94, CI: 1.01–3.75), habit of alcohol (AOR = 8.46, CI: 5.28–13.55) friends' history of alcohol (AOR = 2.16, CI: 1.19–3.94) and intimate partners' history of alcohol (AOR = 2.16, CI: 1.30–3.75) were positively associated with AUD.

**Conclusions:**

Nearly one-third of the respondents' experiences AUD, with factors including age, sex, ethnicity, residential status, religion, family type, education, occupation, food sufficiency from own land, personal alcohol habits, and social connections. Hence, this study recommends screening and treatment in primary healthcare, emphasizing government orientation for healthcare workers.

## 1. Introduction

Excessive alcohol consumption is a significant global public health problem [1]. Alcohol use disorder (AUD), as defined by the diagnostic and statistical manual of mental disorders (DSM-5), is characterized by compulsive alcohol use, loss of control, tolerance, and withdrawal symptoms within a 12-month period [2]. Globally, AUD affects approximately 400 million individuals, with 209 million, equivalent to 3.7% of the world's adult population, experiencing alcohol dependence. [3]. A 2020 cross-national analysis from the World Mental Health Surveys reported a lifetime prevalence of AUD was found to be 8.6%, while the 12-month prevalence was 2.2%. Among individuals who were not abstainers, the lifetime rate increased to 10.7%, and the past-year rate rose to 4.4% [4]. In 2016, AUD was responsible for about 5.3% of worldwide fatalities and 5.1% of global disability-adjusted life years, outstripping the death toll from major conditions such as HIV/AIDS, *tuberculosis*, and diabetes [5]. The World Health Organization's (WHO) 2024 report highlights that the treatment coverage for AUD remains low worldwide, with only 1%–35% of affected individuals receiving services in 2019, varying by country [3]. Globally, alcohol use is rising, with over 155 million adolescents and 47% of adults identified as current drinkers, and heavy episodic drinking increasing among both groups [6, 7]. It has been found that, the development of dependence and abuse is more likely to occur in those who start substance use at younger age in contrast to those who start later [8].

Alcohol use consistently ranks among the top ten risk factors for disease burden globally [9, 10]. Research across five low- and middle-income countries revealed substantial deficiencies in detecting and treating AUD among adult primary care patients [11]. A study conducted at primary healthcare facilities in northern rural Tanzania reported an AUD prevalence of 23.9% [12]. The research found that 18.5% of participants attending primary healthcare facilities had AUDs, with social determinants significantly influencing alcohol consumption behaviors [13]. In the WHO South-East Asia Region, alcohol consumption contributes to health risks among its 1.9 billion people (29% of the global population) [14]. AUD can manifest in various forms, including harmful use, hazardous use, and dependence [15]. It increases the elevated risk of various adverse outcomes, encompassing injuries, hypertension, stroke burden of diseases and mortalities [15–21], and significant financial burdens [22, 23]. In Nepal, alcohol-related challenges are evident, yet few studies have been conducted. The 2013 WHO STEPS survey conducted in Nepal reported that 17% of the surveyed population (28% male and 7.1% female) consumed alcohol in the last 30 days, with 18.6% of males and 2.9% of females engaging in binge drinking [24]. Population-based surveys conducted among adults in the Chitwan district, Nepal, from 2013 to 2017, reported that 15%–57% had consumed alcohol at least once, while 1.5%–25% met criteria for AUD [25]. A study conducted in urban Nepal reported that 20.3% of adults screened positive for AUD using the AUD identification test (AUDIT) tool, underscoring a substantial local burden [26]. In Nepal, alcohol is identified as the eleventh most significant contributor to Disability Adjusted Life Years (DALYs) [24].

The occurrence of AUD is multifactorial, involving a range of associated factors. Globally, research identifies higher household income, older age, marital status, gender, and higher education was associated with AUD [4]. Children who resided in a stable, two-parent household had a reduced likelihood of developing AUD compared to those who never experienced such a family structure [27]. Similarly, another study found that unmarried status, religious practices, and urban residence were significantly linked to AUDs [28]. Studies conducted in Nepal suggest that peer pressure, family environment, and socioeconomic factors exacerbate alcohol misuse [29], while studies in urban areas like Dharan, Nepal, link peer pressure and bad relationships with family and social networks to elevated rates of alcohol dependence [30]. The study conducted in Pokhara, Nepal, found that gender, ethnic group, educational status, and occupation are significantly associated with alcohol consumption patterns [31]. Similarly, another study conducted in primary healthcare services in Nepal found rates of AUD were lower among females and varied by education, caste/ethnicity, occupation, and family income [32]. Although AUD is a recognized as complex, limited research has been conducted to find its prevalence and associated factors in Nepal. Despite AUD's recognized global and national burden, gaps persist in identifying its factors, particularly in resource-limited settings like Nepal's primary healthcare facilities. Early screening and intervention in such settings could substantially reduce its burden, yet studies targeting these populations remain insufficiently explored [32]. This research gap is also found in the Rupandehi district. Hence, this study aims to determine the prevalence and factors associated with AUD among people attending primary healthcare facilities in the Rupandehi district, Nepal.

## 2. Materials and Methods

### 2.1. Study Setting

Nepal, situated in South Asia, is a landlocked country in the southern Himalayas, sharing borders with China and India. It occupies an area of 147,181 km^2^, located between 26°22′–30°27′ north latitude and 80°4′–88°12′ east longitude. Following the adoption of a new constitution in 2015, the country has been reclassified into seven provinces and 77 districts [33]. In Nepal, public health facilities are broadly classified into three levels: primary, secondary, and tertiary. At the primary level, facilities offer promotive and preventive services, alongside basic curative care. At this level, female community health volunteers (FCHVs), primary healthcare outreach clinics, health posts, urban health centers and primary healthcare centers (PHCs) are involved in service delivery. Medical officers, health assistants, staff nurses, auxiliary health workers, auxiliary nurse midwives, and FCHVs play crucial roles in offering services [34, 35]. Secondary-level facilities act as referral centers for primary care, offering additional services such as inpatient and outpatient treatment, emergency care, and comprehensive obstetric and neonatal emergency services. Tertiary-level facilities, in turn, serve as referral hubs for secondary care, providing advanced support to lower-level facilities, along with professional training and research activities [34]. Typically, primary and secondary healthcare services serve most individuals' health needs; however, severe injuries or complex conditions necessitate the specialized care available at higher-level facilities [36]. Rupandehi district is one of the 12 districts in Nepal's Lumbini province. It is bounded on the east by Nawalparasi, on the west by Kapilvastu, on the north by Palpa, and on the south by Uttar Pradesh, India. The district covers a total area of 141,340 ha and is located at an altitude of 95 m–1219 m above sea level, this district is divided into 3 geographical regions including Chure region, Bhawar region, and Terai/Madhes [37]. According to the census 2021, the district has 238,171 households and a population of 1,121,957 out of which 550,478 are males and 571,479 are females [38]. In Rupandehi district, there are four government hospitals, twenty-one private hospitals, five PHCs, sixty-two health posts, fifty-four basic healthcare centers, two community health units, and 12 urban health centers. The primary healthcare facilities include PHCs, health posts, basic healthcare centers, community health units, and urban health centers [39].

### 2.2. Study Design, Period, and Population

An institutional-based cross-sectional study was conducted at primary healthcare facilities in the Rupandehi district of Nepal from September 2021 to February 2022. The required data for this study was collected from primary sources. Individuals aged 18 years and older who visited primary healthcare facilities in the Rupandehi district, Nepal, for healthcare services were included in the study. The people having an age of less than 18 years, suffering from severe medical problems at the time of interview rendering them unfit for interview, and who did not provide written informed consent were excluded from the study.

### 2.3. Sample Size and Sampling Technique

The sample size of the study was 688 which was calculated by using the formula: *n* = Z^2^pq/L2 [40] where *Z* is the standard normal deviate set for a 95% confidence level (1.96), *p*=0.073 is the prevalence of AUD among adults attending primary healthcare services in Nepal was 7.3% [32], *q* is the 1-p (0.927), and *L* is the margin of error (2.5%). The initial calculation yielded a sample size of 416: *n*= (1.96)^2^ × (0.073) × (0.927)/(0.025)^2^ = 416. Since the study employed a multistage probability random sampling technique, the calculated sample size of 416 was adjusted by applying a design effect of 1.5. This value aligns with the Nepal Demographic and Health Survey (NDHS) 2022 (Appendix B, p. 578), which reports a design effect of 1.494 for current tobacco use in Lumbini Province using a similar multi-stage probability sampling technique [35] and in the previous study on maternal alcohol consumption conducted in Ethiopia [41]. Thus, the sample size (*n*) became 416 × 1.5 = 624. To account for potential non-responses, a common challenge in primary healthcare settings where participation may be incomplete, the calculated sample size of 624 was increased by a 10% nonresponse rate (to 687, that is, 624 + 63). This common adjustment aligns with standard practice in cross-sectional studies to ensure sufficient data when exact nonresponse rates are unavailable [42] and was used in an alcohol consumption-related study in Gondar town, Ethiopia [41]. For the alignment of sampling technique involving an equal number of respondents from each of 8 health facilities, the sample size was rounded to 688. Hence, the final sample size was 688.

**Figure 1:**
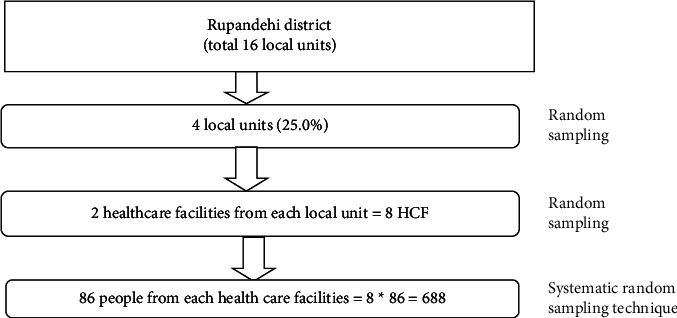
Sampling technique.

The Rupandehi district comprises 16 local units, from which four—Butwal submetropolitan city, Siddharthanagar municipality, Mayadevi rural municipality, and Sudhodhan rural municipality—were randomly selected. From each of these two primary healthcare facilities, four were randomly selected, resulting in a total of eight facilities. A systematic sampling technique was employed to select respondents, achieving a total sample size of 688 by enrolling an equal number of 86 individuals from each of the eight primary healthcare facilities (8 × 86 = 688) (Figure 1). Due to the unavailability of exact data on the number of patients visiting each healthcare facility, an equal number of 86 respondents were selected from each facility to ensure consistency and simplicity in the sampling process, an approach supported in studies addressing populations with incomplete records [43–45]. One trained enumerator was assigned to each facility, and every third respondent visiting the facility was interviewed, after skipping the preceding two respondents, until the target was reached. When multiple individuals were present simultaneously, one individual was randomly selected to ensure fairness. Data were collected from the respondents through face-to-face interviews. ‘Primary-level health facilities were targeted because they serve as the first point of contact for most residents, particularly in rural settings, and are critical for early AUD screening and intervention, aligning with the study's focus on primary care settings.

### 2.4. Instrument and Validity

An questionnaire regarding sociodemographic and socioeconomic as well as exposure to alcohol use was formulated by reviewing different literatures [12, 46–48] and used for obtaining information on various factors associated with AUD. To assess exposure to alcohol use, we included specific questions on participants' regular personal habit of alcohol consumption (e.g., “Do you regularly consume alcohol?”) and the alcohol consumption history of their friends (e.g., “Do your friends consume alcohol?”), intimate partners (e.g., “Does your intimate partner consume alcohol?”), similarly alcohol consumption by father, mother, siblings, and other family members. These questions, adapted to capture social influences on alcohol use, were administered during face-to-face interviews with binary (yes/no) responses [49–51]. Participants provided binary responses (yes/no) to these questions during face-to-face interviews. The formulated questionnaire was translated into Nepali and again retranslated in English language for the consistency. For this study, educational levels were categorized as follows: Illiterate (unable to read and write), basic literacy (able to read and write minimal text, such as name), primary (grades 1–5), lower secondary education (class 6 to class 8), secondary education (classes 9 and 10) based on the Nepal's former education system, and SLC/SEE and higher combined into a single category, consistent with prior studies [52, 53].

To assess AUD, the study utilized the validated AUDIT instrument in its Nepali version. WHO developed the AUDIT as a screening tool for AUD in primary healthcare [54]. The AUDIT has been validated across diverse populations in low- and middle-income countries, affirming its reliability and applicability in various settings [55–57]. The validation of the AUDIT in Nepal was conducted utilizing DSM Fourth Edition (DSM-IV) diagnostic categories as the gold standard for determining diagnostic parameters. A recommended cutoff score of ≥ 9 has been proposed for both males and females. For males, the recommended cutoff manifests a sensitivity of 96.7%, specificity of 91.7%, a positive predictive value (PPV) of 90.3%, and a negative predictive value (NPV) of 97.2% in the identification of alcohol dependence or abuse. Likewise, for females, the suggested cutoff demonstrates a sensitivity of 94.3%, a specificity of 91.4%, a PPV of 80.1%, and an NPV of 97.8% for the same purpose [56]. AUDIT has demonstrated good reliability in Nepal, with a Cronbach's alpha of 0.82 [56], and was used without additional pilot testing due to its established applicability in this context.

### 2.5. Data Collection

Eight trained enumerators with undergraduate medical qualifications conducted face-to-face interviews with the respondents. They were actively involved in data collection and field coordination, under the close supervision of the principal investigator. The enumerators underwent a comprehensive six-day training program led by the principal investigator, which covered data collection techniques, probing methods; skip patterns, and ethical considerations. The principal investigator ensured that the enumerators fully understood the directions for administering the AUDIT scale through interactive training sessions. During the six-day program, the enumerators participated in hands-on practice, role-playing exercises, and demonstrations to reinforce the correct administration of the AUDIT scale. The principal investigator also conducted practical assessments, including mock interviews, to evaluate their comprehension and proficiency in administering the AUDIT scale correctly before beginning data collection. This ensured that all enumerators were well-prepared and confident in their roles. Interviews were conducted outside the outpatient department (OPD) rooms or within the premises of the primary healthcare facilities. The collected data was regularly reviewed and edited by the principal investigator to ensure completeness and consistency.

### 2.6. Measurement of AUD and AUDIT Zone Category

The validated Nepali version of the AUDIT was used to measure the prevalence of AUD at a cut-off point of ≥ 9 [56]. AUDIT served as a guiding tool to ascertain the risk level category. This classification system delineates AUDIT scores as follows: a score of 0–7 corresponds to Zone I for low-risk drinking, a score of 8–15 designates Zone II for higher-risk drinking, a score of 16–19 signifies Zone III for harmful and hazardous drinking, and a score of 20–40 identifies Zone IV for possible alcohol dependence among adults, based on their drinking status [58].

### 2.7. Data Processing and Analysis

Collected data underwent a cross-check to ensure all the questions were filled correctly. The researcher manually checked, compiled, and edited the data. The data were then entered into Microsoft Excel and analyzed using Statistical Package for the Social Sciences (SPSS) version 22. In descriptive statistics frequency, percentage, median, and quartile were calculated, and in inferential statistics, bivariate analysis was conducted to find the association between AUD with various independent variables using the chi-square test, considering a *p* value of < 0.05 as significant. Variables which were found to be significantly associated in bivariate analysis with *p* value less than 0.05 were adjusted in multivariate analysis to determine the net effect of independent variables on AUD. This was done after checking for multicollinearity (Variance Inflation Factor [VIF] < 10) and using Nagelkerke *R* square via binary logistic regression as in the previous study [59]. The analysis of background-related factors revealed a Nagelkerke *R* square value of 0.384, indicating that 38.4% of the variation in AUD was explained by the significant independent variables. The VIF of all significant independent variables ranged from 1.048–1.978 (less than 10), indicating no multicollinearity. The data were summarized, adjusted odds ratios (AORs) were estimated, and their corresponding values at 95% confidence intervals were computed. Similarly, the analysis of alcohol-related factors revealed a Nagelkerke *R* square value of 0.420, indicating that 42.0% of the variation in AUD was explained by the significant independent variables. The VIF of all significant variables ranged from 1.162–2.023 (less than 10); indicating no multicollinearity among the independent variables.

### 2.8. Ethical Approval and Consent to Participate

Ethical approval for this study was obtained from the Institutional Review Committee of Universal College of Medical Science and Teaching Hospital (UCMS/IRC/096/21), Bhairahawa Rupandehi, Nepal. Official letters were sent to the relevant stakeholders of local units and health facilities, and formal permission was obtained through the appropriate channels. All the participants were fully informed about the nature of the study, its objectives, and the confidentiality of the collected information. Only those respondents who voluntarily agreed to participate were involved in the study. Written informed consent was obtained from each respondent, while thumbprints were taken from those who were illiterate. The confidentiality and privacy of the information were maintained throughout the study. Respondents' dignity was respected by providing right to reject or discontinue participation in the research study at any point.

## 3. Results

In order to achieve the objective of the study, a cross-sectional survey was conducted with 688 participants, utilizing the validated Nepali version of the AUDIT to screen for AUD. Data were analyzed using bivariate and multivariate logistic regression to determine significant associations. This section presents the findings, beginning with the background characteristics of the study population, followed by the prevalence of AUD, its distribution across AUDIT risk categories, and the factors significantly associated with AUD. The median age of the respondents was 30 years (Q1–Q3: 23–42). More than one-fourth (27.9%) of the respondents were female, with Brahmin/Chhetri (31.1%) and Madeshi (30.1%) being the largest ethnic groups. Nearly half (45.5%) resided in rural municipalities, and 91.6% were Hindus. More than two-thirds (67.3%) were married, with a median family size of five members (Q1–Q3: 4–7). Over half (52.6%) resided in joint families, and nearly half (48.3%) lived with both parents. In terms of education, 12.8% were illiterate, while 8.9% had basic literacy. Occupationally, 11.8% of the respondents were homemakers, and about one-third (30.1%) engaged in small-scale business. Food sufficiency for nine to 12 months or more from their own land was reported by less than half (44.2%) (Table 1).

Alcohol consumption was reported by more than half of the respondents' fathers (52.2%) and by 10.3% of their mothers. Siblings' alcohol use was reported by 41.6% and 29.4% of other family members consumed alcohol. Nearly half (44.2%) of the respondents have regular personal habit of alcohol consumption, with 62.5% having friends and 50.6% having intimate partners with a history of alcohol consumption (Table 2).

Approximately one-third (30.8%, 95% CI:27.4–34.4) of the respondents had AUD. Of the respondents, 62.8% were classified in Zone I (low-risk drinking), 26.7% in Zone II (higher-risk drinking), 5.1% in Zone III (harmful and hazardous drinking), and 5.4% in Zone IV (possible alcohol dependence) (Table 3).

Table 4 represents background-related factors associated with AUD. The multivariate regression analysis model revealed that age, sex, caste/ethnicity, residential status, religion, type of family, education, occupation, and food sufficiency from own land were identified as associated factors with AUD among the respondents. The odds of experiencing AUD were 45% less likely among respondents in the age groups of 20–29 (AOR = 0.45, CI: 0.24–0.86) and 26% less likely among those aged ≥ 50 years (AOR = 0.26, CI: 0.11–0.61), compared to individuals aged ≤ 19 years. Females were 14% less likely (AOR = 0.14, CI: 0.07–0.28) to experience AUD than their counterparts. Newar were 4 times more likely (AOR = 4.10, CI: 1.00–16.88) to experience AUD than Brahman and Chhetri. Respondents living in the rural area (rural municipalities) were 1.57 times more likely (AOR = 1.57, CI: 1.02–2.42) to experience AUD than those who were living in urban areas (submetropolitan cities and municipalities). Those respondents who followed regions other than Hindu religion were 5% less likely (AOR = 0.05, CI: 0.01–0.43) to experience AUD than their counterparts. Respondents who were living in joint family were 1.58 times more likely (AOR = 1.58, CI: 1.05–2.37) to experience AUD than those living in nuclear family. The odds of experiencing AUD were 20% less likely among respondents with primary education (AOR = 0.20, CI: 0.09–0.43), 16% less likely among those with lower secondary education (AOR = 0.16, CI: 0.07–0.38), 21% less likely among those with secondary education (AOR = 0.21, CI: 0.10–0.47), and 16% less likely for those with SLC and above education (AOR = 0.16, CI: 0.08–0.31), in comparison to individuals who were illiterate. Respondents engaged in business work were 2.91 times more likely (AOR = 2.91, CI: 1.00–8.48) to experience AUD compared to homemakers. Those working in services were 3.30 times more likely (AOR = 3.30, CI: 1.08–10.12). Individuals with daily wage occupations had a 3.57 times higher likelihood (AOR = 3.57, CI: 1.10–11.56), while those involved in agriculture were 4.25 times more likely (AOR = 4.25, CI: 1.36–13.31), to experience AUD, compared to homemakers. Respondents with 6–9 months of food sufficiency from their own land were 1.94 times more likely (AOR = 1.94, CI: 1.01–3.75) to experience AUD than those with less than 3 months of food sufficiency (Table 4).

The multivariate regression analysis model revealed that the variables including personal habit of alcohol consumption, friends' history of alcohol consumption, and intimate partners' history of alcohol consumption were identified as associated factors with AUD among the respondents. The respondents who reported having regular personal habit of alcohol consumption were 8.46 times more likely (AOR = 8.46, CI: 5.28–13.55) to experience AUD compared to their counterparts. The respondents whose friends have history of alcohol consumption were 2.16 times more likely (AOR = 2.16, CI: 1.19–3.94) to experience AUD compared to their counterparts. Similarly, the respondents whose intimate partners' have history of alcohol consumption were 2.20 times more likely (AOR = 2.16, CI: 1.30–3.75) to experience AUD compared to their counterparts (Table 5).

Nearly one-third of the respondents had AUD, with most falling into the low-risk category (62.8%), though a notable portion were at higher risk (26.7%), and smaller groups showed harmful drinking (5.1%) or possible dependence (5.4%). Factors like age, gender, ethnicity, rural living, education, occupation, and food sufficiency played a role, alongside personal drinking habits and the influence of friends and partners who drink.

## 4. Discussion

The prevalence of AUD was found to be nearly one-third, highlighting a significant public health concern [56]. Several background-related factors including age, sex, caste/ethnicity, residential status, religion, type of family, education, occupation, and food sufficiency from own land, along with exposure-related factors such as regular personal alcohol consumption habit, friends' history of alcohol consumption, and intimate partners' history of alcohol consumption, were identified as associated factors with AUD among the respondents. These results contribute to understanding AUD in the context of primary healthcare facilities in Rupandehi District, Nepal, they also reveal gaps when compared to previous studies. Specifically, the prevalence and associated factors in this study differ from some prior research in Nepal and globally, highlighting a lack of consistent data on AUD across diverse populations and settings within Nepal. These gaps suggest a need for more localized studies to explore contextual differences in AUD prevalence and associated factors, especially in rural primary care settings where data remain limited.

The finding of this study revealed that nearly one-third (30.81%) of the respondents experienced AUD which is in line with several other studies, such as those conducted in Ethiopia [47, 60], among people living with HIV in Ethiopia [61], Lebanon [62] and among Jimma University undergraduate students [63]. However, in contrast to this study, several other studies conducted in Nepal [25, 46, 64], India [65], Brazil [66], Russia [67], Ethiopia [28], and rural Tanzania [12] found a lower proportion of respondents experience AUD. The variations in the prevalence of AUD observed across these studies could be attributed to differences in study populations (e.g., general vs. specific groups like students or HIV patients), settings, diagnostic tools (e.g., AUDIT vs. other criteria), and timeframes. Socioeconomic factors such as education, occupation, and food sufficiency, and sociodemographic variables such as age, sex, caste, residential status, religion, and type of family which were measured and adjusted for in multivariate analysis (Table 4) also contribute to these differences. Additionally, cultural factors, such as societal norms around alcohol use mentioned in the Introduction, may play a role, though they were not directly assessed in this study due to its quantitative focus. In Nepal's context, Rupandehi's mixed urban-rural setting and social acceptance of drinking in certain communities might elevate prevalence compared to more conservative regions. A potential solution is to implement community-based screening programs tailored to local drinking norms, enhancing early detection and intervention in primary care facilities.

This study revealed that approximately two-thirds of the respondents were categorized as low-risk drinkers (Zone I), aligning with findings from other studies such as study conducted in West Bengal, India [68], and Sehore district, India [69]. However, a lower proportion was characterized into the low-risk category in another study conducted Michigan [70]. In contrast to this study, a study conducted in England found a higher proportion of the respondents were categorized into the low-risk category [71]. Similarly, more than one-fourth of the participants in this study were categorized into the higher-risk drinking category (Zone II), consistent with the findings from other studies conducted in the Sehore district India [69] and Michigan [70]. However, this study is dissimilar from the results of studies conducted in West Bengal, India [68], and in England [71]. Regarding the patterns indicative of harmful and hazardous drinking (Zone III) and possibly having alcohol dependence (Zone IV), the study found that 5.1% exhibit patterns indicative of harmful and hazardous drinking (Zone III), while the remaining 5.4% were identified as possibly having alcohol dependence (Zone IV), according to the AUDIT-risk level categories. These findings align with the previous study conducted in West Bengal and Sehore district India [68, 69] and among people living with HIV in Ethiopia [61]. In contrast to this study, several other studies such as studies conducted in primary healthcare facilities in Brazil, found higher portion classified as abstemious or low-risk users (level I) and a lower portion were classified as the higher-risk drinking (Zone II), harmful and hazardous drinking (Zone III), and having alcohol dependence (Zone IV) categories among the respondents [72]. This inconsistency might be linked to differences in alcohol policies, access to alcohol, study populations, settings, timeframes, and social and cultural aspects of drinking. Locally, integrating AUD education into primary care and regulating alcohol sales could mitigate these patterns.

Numerous sociodemographic, socioeconomic, and exposure-related variables were found to be significantly associated with AUD. In this study, age of the respondents was significantly associated with AUD, aligning with several previous studies such as studies conducted in the central district of Nepal [25], among the youth of Suryabinayak Municipality, Bhaktapur, Nepal [73], Ethiopia [60], Brazil [66, 72], Russia [67] and Georgia [74]. Older age groups may face exposure or stress-related drinking triggers. In Rupandehi, targeting older adults with peer-support programs could address this gap. In the present study, females were found less likely to experience AUD. This finding is consistent with results from the previous studies conducted in Chitwan, Nepal [32, 64], Lebanon [75], among people living with HIV in Ethiopia [61], Hawassa city, Ethiopia [60], Gondar, Ethiopia [47], Brazil [66, 76], northern Tanzania [1], and in Russia [67]. Gender-specific awareness campaigns could reinforce prevention among women while addressing male drinking culture. Caste/ethnicity was identified as significantly associated with AUD in this study, revealing that individuals outside the Brahman/Chhetri caste were more likely to experience AUD. This finding is consistent with previous studies conducted in the central district of Nepal [25], the Chitwan district of Nepal [64], and Jimma University Specialized Hospital [61] where individuals from disadvantaged castes were more likely to experience AUD. Social marginalization or traditional alcohol use in certain ethnic groups (e.g., Dalits or Janajatis) might explain this, differing from studies lacking caste focus. Community-based interventions engaging ethnic leaders could reduce AUD in these groups locally. The present study indicated a higher likelihood of AUD among respondents residing in rural areas. In contrast to these findings, a previous study conducted in Goa, India [77] found that AUD was more prevalent in urban areas. Another study conducted in rural India reported a positive association between AUDIT scores and urban residence [69]. Similarly, a study within a community in Ethiopia yielded results contradictory to the present study [28]. This discrepancy might be associated with social and cultural aspects of drinking. Rural isolation, poverty, or traditional brewing in Rupandehi might drive this, unlike urban-centric studies. Enhancing rural healthcare access and economic opportunities could address this locally. Religion was found to be significantly associated in the present study, revealing that individuals practicing non-Hindu religions were less likely to have AUD. This association aligns with findings from other studies conducted in the central district of Nepal [25], Jimma University Specialized Hospital [61], and Ethiopia [28], as well as Northern Tanzania [1]. Type of family was found to be significantly associated with AUD in this study. However, another study conducted in Korea reported dissimilar results, as family type was not statistically significant with AUD [78]. This discrepancy could be attributed to variations in the study population, differences in the study settings, as well as the socioeconomic and cultural factors of the respondents. Joint families in Nepal might enable drinking through social reinforcement, differing from nuclear family. Family counseling focused to joint family structures could mitigate this in the local setting. The present study revealed that higher education was associated with lower odds of having AUD. This finding is consistent with several other studies, including those conducted in Chitwan district, Nepal [25, 64], southern Brazil [76], England [79] and Denmark [80]. In the present study, occupation of the respondents was found to be significantly associated with AUD, aligning with the findings of previous studies conducted in the central district of Nepal [25], Chitwan, Nepal [64] and West Bengal, India [68]. The present study revealed that individuals with food sufficiency from their own land for a period of 6–9 months were more likely to experience AUD compared to those with sufficiency for equal to or less than 3 months. This finding aligns with a study conducted in Bhutan, where respondents with higher incomes were more likely to engage in binge drinking than those with low incomes [81]. However, a study conducted in rural India reported a negative association between AUDIT scores and individuals having their own land [69]. In contrast to this study, another study conducted in Chitwan, Nepal [64], found that family income sufficient to manage foods for the period of 6–9 months was not statistically significant in relation to AUD. This disparity might be attributed to the differences in socioeconomic as well as cultural factors between the two settings. Nutrition education tied to AUD prevention could be a local solution. The current study revealed that individuals with a regular habit of alcohol consumption were more likely to experience AUD. This finding is in line with another study conducted in southern Ethiopia [82], which reported that individuals with a lifetime history of substance use were more likely to have AUD. In the present study, individuals with a history of alcohol use among friends were more likely to experience AUD. Similar results were also found in other studies conducted among the youth of Suryabinayak Municipality, Bhaktapur, Nepal [73], and in Jimma University undergraduate students [63], where AUD was significantly associated with respondents having friends with a history of alcohol use and experiencing peer pressure to drink alcohol. Similarly, another study conducted among college students in Bhutan found that respondents whose close friends used alcohol were more likely to be binge drinkers than those who had close friends who did not use alcohol [81]. The present study revealed that individuals with an intimate partner history of alcohol use were more likely to experience AUD. Similar results were also found in several other studies, such as studies conducted in southern Ethiopia [82] and Sweden [83], which found that the risk for AUD was substantially increased when the partner had an AUD. Peer and partner influence might reinforce drinking habits. Peer and couple-based counseling in primary care could address this.

## 5. Conclusions

Nearly one-third of the respondents in the present study were found to have AUD, with 26.7% in Zone II (higher-risk drinking), 5.1% in Zone III (harmful and hazardous drinking) and 5.4% in Zone IV (possible alcohol dependence). While there is no universally defined threshold for an ‘acceptable' prevalence of AUD below which intervention is unnecessary, this level particularly with over 10% in harmful, hazardous, or dependent categories suggests a significant public health concern, especially in a resource-limited setting like Nepal. Screening and treating individuals with AUD in primary healthcare facilities have been recommended as effective strategies to minimize the treatment gap. Therefore, the government of Nepal should consider providing orientation and training programs to healthcare workers in such facilities.

Age, sex, caste/ethnicity, residential status, religion, type of family, education, occupation and food sufficiency from own land, personal regular habit of alcohol consumption, friend history of alcohol consumption and intimate partners' history of alcohol consumption were identified as associated factors with AUD among the respondents. Based on this finding, this study suggests the need for cooperative and immediate attention from governmental and nongovernmental organizations, including multisectoral responses from health, education and local governance sectors. It is recommended that the local government prioritize awareness campaigns, community sensitization, and peer support programs, especially for teenagers, males, lower-caste individuals, and rural populations, to show that alcohol habits are modifiable and controllable. Similarly, tax regulation should be increased in alcoholic products to reduce the AUD [84–87]. Likewise, it is advisable to endorse comprehensive community-based research utilizing qualitative approaches to explore perceptions, cultural practices, beliefs, and value systems associated with alcohol use.

## 6. Strengths and Limitations of the Study

### 6.1. Limitation of the Study

It is important to acknowledge that this study has certain limitations that may affect the validity of the findings. Due to the cross-sectional nature of the study, the causal inference cannot be made, as both the determinants and outcomes were assessed concurrently. The study focused on adults aged 18 years and above, excluding children and adolescents, a key population receiving services from primary healthcare facilities in Nepal, thus limiting insights into their alcohol use and mental health. Potential information bias may arise from reliance on respondents' recall. Another limitation is that cultural factors such as norms, beliefs, or practices related to alcohol use, which may influence AUD prevalence, were not directly measured or controlled for in this study due to its quantitative design and focus on sociodemographic and exposure-related variables. Further research should employ longitudinal designs to establish causality, include younger populations to broaden the scope, validate findings with objective measures to reduce recall bias, and explore cultural influences on AUD to enhance contextual understanding.

### 6.2. Strengths of the Study

This study possesses a notable strength as it was conducted within a primary healthcare setting, an environment where information on AUD is notably scared. Additionally, it adopted a population-based approach, conducting interviews in real-life settings, which enhances the authenticity and representativeness of the findings.

## Figures and Tables

**Table 1:** Distribution of background related characteristics of study population.

General characteristics		Frequency *n* = 688 (percentage)
Age of respondents	≤ 19 years	69 (10)
	20–29 years	268 (39)
	30–39 years	143 (20.8)
	40–49 years	102 (14.8)
	≥ 50 years	106 (15.4)
	Median age of respondents in years (Q_1_–Q_3_); 30 (23–42)	

Sex of respondents	Male	496 (72.1)
	Female	192 (27.9)

Caste/Ethnicity	Brahman/Chhetri	214 (31.1)
	Madeshi	207 (30.1)
	Dalits	57 (8.3)
	Newar	13 (1.9)
	Janjati	140 (20.3)
	Muslim	45 (6.5)
	Others	12 (1.7)

Residential status	Urban	375 (54.5)
	Rural	313 (45.5)

Religion	Hindu	630 (91.6)
	Others	58 (8.4)

Marital status	Unmarried	207 (30.1)
	Married	463 (67.3)
	Widow/widower/separated	15 (2.2)
	Divorce	3 (0.4)

Size of family	≤ 4 members	231 (33.6)
	> 4 members	457 (66.4)
	Median family size (Q_1_–Q_3_); 5 (4–7)	

Type of family	Nuclear	279 (40.6)
	Joint	362 (52.6)
	Extended	47 (6.8)

Living status	Both parents	332 (48.3)
	Mothers only	83 (12.1)
	Relatives	27 (3.9)
	Others	246 (35.8)

Education	Illiterate (unable to read and write)	88 (12.8)
	Basic literacy(able to read and write)	61 (8.9)
	Primary (1-5 class)	84 (12.2)
	Lower secondary (6-8 class)	66 (9.6)
	Secondary (9-10 class)	94 (13.7)
	SLC/SEE and above	295 (42.9)

Occupation	Home maker	81 (11.8)
	Business	207 (30.1)
	Services	139 (20.2)
	Daily wages	63 (9.2)
	Agriculture	72 (10.5)
	Students	126 (18.3)

Earning cash money	Not earning	14 (2)
	Earning	674 (98)

Family income sufficient to arrange food for period of	≤ 3 months	232 (33.7)
	3–6 months	77 (11.2)
	6–9 months	75 (10.9)
	≥ 9–12 months	304 (44.2)

Abbreviations: SEE, secondary education examination; SLC, school leaving certificate.

**Table 2:** Distribution of parental, siblings, and friends' alcohol consumption status.

General characteristics		Frequency *n* = 688 (percentage)
Alcohol consumption by father	No	359 (52.2)
	Yes	329 (47.8)

Alcohol consumption by mother	No	617 (89.7)
	Yes	71 (10.3)

Alcohol consumption by siblings	No	402 (58.4)
	Yes	286 (41.6)

Alcohol consumption by other family members	No	486 (70.6)
	Yes	202 (29.4)

Regular habit of alcohol consumption	No	384 (55.8)
	Yes	304 (44.2)

Friend history of alcohol consumption	No	258 (37.5)
	Yes	430 (62.5)

Intimate partner friend history of alcohol consumption	No	340 (49.4)
	Yes	348 (50.6)

**Table 3:** Prevalence of alcohol use disorder and distribution of respondents according to the WHO AUDIT-risk level category.

**Characteristics**		**Frequency *n* = 688 (percentage)**	**95% confidence interval (95% CI)**

Prevalence of alcohol use disorder		**212 (30.81)**	**27.4–34.4**

**AUDIT-risk level**	**AUDIT score**		

Zone I (low risk drinking)	0–7	432 (62.8)	59.1–66.4
Zone II (higher risk drinking)	8–15	184 (26.7)	23.5–30.2
Zone III (harmful and hazardous drinking)	16–19	35 (5.1)	3.6–7.0
Zone IV (possible alcohol dependence)	20–40	37 (5.4)	3.8–7.3

*Note:* Bold value denotes highlighting the overall prevalence of AUD with its confidence interval at 95%.

**Table 4:** Background related factors associated with alcohol use disorder in using bivariate and multivariate analysis.

Characteristics	Alcohol use disorders (%)		*p* value	COR	95% CI	AOR	95% CI	*p* value
	No AUD (*n* = 476)	AUD (*n* = 212)						
*Age of respondents*								
≤ 19 years	37 (53.6)	32 (46.4)	0.025^∗^	1		1		
20–29 years	194 (72.4)	74 (27.6)		0.44	**0.26–0.76**	0.45	**0.24–0.86**	**0.015**
30–39 years	99 (69.2)	44 (30.8)		0.51	**0.28–0.93**	0.55	0.25–1.19	0.126
40–49 years	67 (65.7)	35 (34.3)		0.60	0.32–1.13	0.74	0.32–1.68	0.466
≥ 50 years	79 (74.5)	27 (25.5)		0.40	**0.21–0.75**	0.26	**0.11–0.61**	**0.002**

*Sex of respondents*								
Male	299 (60.3)	197 (39.7)	< 0.001^∗^	1		1		
Female	177 (92.2)	15 (7.8)		0.13	**0.07–0.22**	0.14	**0.07–0.28**	**< 0.001**

*Caste/Ethnicity*								
Brahman/Chhetri	166 (77.6)	48 (22.4)	0.033^∗^	1				
Madeshi	136 (65.7)	71 (34.3)		1.81	**1.17–2.78**	1.04	0.62–1.74	0.875
Dalits	36 (63.2)	21 (36.8)		2.02	**1.08–3.78**	1.01	0.48–2.15	0.975
Newar	7 (53.8)	6 (46.2)		2.96	0.95–9.24	4.10	**1.00–16.88**	**0.049**
Janjati	90 (64.3)	50 (35.7)		1.92	**1.20–3.08**	1.53	0.87–2.68	0.136
Muslim	34 (75.6)	11 (24.4)		1.12	0.53–2.37	9.73	0.92–10.27	0.059
Others	7 (58.3)	5 (41.7)		2.47	0.75–8.13	2.08	0.48–9.00	0.329

*Residential status*								
Urban	280 (74.7)	95 (25.3)	0.001^∗^	1		1	1	
Rural	196 (62.6)	117 (37.4)		1.76	**1.27–2.44**	1.57	**1.02–2.42**	**0.042**

*Religion*								
Hindu	429 (68.1)	201 (31.9)	0.041^∗^	**1**		**1**	**1**	
Other than Hindus	47 (81)	11 (19)		**0.50**	**0.25–0.98**	0.05	**0.01–0.43**	**0.007**

*Marital status*								
Unmarried	149 (72)	58 (28)	0.473	**1**		Ns		
Married	316 (68.3)	147 (31.7)		1.20	0.83–1.72			
Widow/widower/divorce	11 (61.1)	7 (38.9)		1.64	0.60–4.42			

*Size of family*								
≤ 4 members	167 (72.3)	64 (27.7)	0.209	1		Ns		
> 4 members	309 (67.6)	148 (32.4)		1.25	0.88–1.77			

*Type of family*								
Nuclear	202 (72.4)	77 (27.6)	0.013^∗^	1		1		
Joint	235 (64.9)	127 (35.1)		1.42	**1.01–1.99**	1.58	**1.05–2.37**	**0.028**
Extended	39 (83)	8 (17)		0.54	0.24–1.20	0.45	0.18–1.14	0.092

*Living status*								
Both parents	226 (68.1)	106 (31.9)	0.359	1		Ns		
Mothers only	63 (75.9)	20 (24.1)		0.68	0.39–1.18			
Relatives	21 (77.8)	6 (22.2)		0.61	0.24–1.55			
Others	166 (67.5)	80 (32.5)		1.03	0.72–1.46			

*Education*								
Illiterate	43 (48.9)	45 (51.1)	0.001^∗^	1		1		
Informal class	43 (70.5)	18 (29.5)		0.40	**0.20–0.80**	0.48	0.20–1.16	0.102
Primary	56 (66.7)	28 (33.3)		0.48	**0.26–0.89**	0.20	**0.09–0.43**	**< 0.001**
Lower secondary	46 (69.7)	20 (30.3)		0.42	**0.21–0.81**	0.16	**0.07–0.38**	**< 0.001**
Secondary	67 (71.3)	27 (28.7)		0.39	**0.21–0.71**	0.21	**0.10–0.47**	**< 0.001**
SLC/SEE and above	221 (74.9)	74 (25.1)		0.32	**0.20–0.52**	0.16	**0.08–0.31**	**< 0.001**

*Occupation*								
Home maker	75 (92.6)	6 (7.4)	< 0.001^∗^	1		1		
Business	147 (71)	60 (29)		5.10	**2.11–12.35**	2.91	**1.00–8.48**	**0.049**
Services	88 (63.3)	51 (36.7)		7.24	**2.95–17.82**	3.30	**1.08–10.12**	**0.037**
Daily wages	35 (55.6)	28 (44.4)		10.00	**3.80–26.35**	3.57	**1.10–11.56**	**0.034**
Agriculture	40 (55.6)	32 (44.4)		10.00	**3.86–25.93**	4.25	**1.36–13.31**	**0.013**
Students	91 (72.2)	35 (27.8)		4.81	**1.92–12.04**	2.26	0.69–7.36	0.177

*Earning cash money*								
Not earning	8 (57.1)	6 (42.9)	0.324	1		Ns		
Earning	468 (69.4)	206 (30.6)		0.59	0.20–1.71			

*Family income to arrange food for*								
≤ 3 months	173 (74.6)	59 (25.4)	0.049	1		1		
3–6 months	51 (66.2)	26 (33.8)		1.50	0.86–2.61	1.27	0.66–2.45	0.474
6–9 months	44 (58.7)	31 (41.3)		2.07	**1.20–3.57**	1.94	**1.01–3.75**	**0.047**
9–12 months or more	208 (68.4)	96 (31.6)		1.35	0.92–1.98	1.18	0.73–1.91	0.511

*Note:* 1 = reference category, Ns, not significant in bivariate analysis.

Abbreviations: AOR, adjusted odds ratio; AUD, alcohol use disorders; CI, confidence interval; COR, crude odds ratio; SEE, secondary education examination; SLC, school leaving certificate.

^∗^Significant at *p* < 0.05. Bold denotes significant.

**Table 5:** Association of exposure-related factors with alcohol use disorder among primary healthcare attendees using bivariate and multivariate analysis.

Characteristics	Alcohol use disorders (%)		*p* value	COR	95% CI	AOR	95% CI	*p* value
	No AUD (*n* = 476)	AUD (*n* = 212)						
*Alcohol consumption by father*								
No	258 (71.9)	101 (28.1)	0.112	1		Ns		
Yes	218 (66.3)	111 (33.7)		1.30	0.94–1.8			

*Alcohol consumption by mother*								
No	429 (69.5)	188 (30.5)	0.565	1		Ns		
Yes	47 (66.2)	24 (33.8)		1.17	0.69–1.96			

*Alcohol consumption by siblings*								
No	305 (75.9)	97 (24.1)	< 0.001^∗^	1		1		
Yes	171 (59.8)	115 (40.2)		2.12	**1.52–2.94**	1.22	0.80–1.86	0.364

*Alcohol consumption by other family members*								
No	349 (71.8)	137 (28.2)	0.021^∗^	1		1		
Yes	127 (62.9)	75 (37.1)		1.50	**1.06–2.13**	0.65	0.41–1.02	0.058

*Regular habit of alcohol consumption*								
No	350 (91.1)	34 (8.9)	< 0.001^∗^	**1**		**1**		
Yes	126 (41.4)	178 (58.6)		14.54	**9.56–22.12**	8.46	**5.28–13.55**	**< 0.001**

*Friend history of alcohol consumption*								
No	235 (91.1)	23 (8.9)	< 0.001^∗^	**1**		**1**		
Yes	241 (56)	189 (44)		8.01	**5.01–12.80**	**2.16**	**1.19–3.94**	**0.012**

*Intimate partner history of alcohol consumption*								
No	302 (88.8)	38 (11.2)	< 0.001^∗^	1		1		
Yes	174 (50.0)	174 (50.0)		7.95	**5.34–11.83**	2.20	**1.30–3.75**	**0.003**

*Note:* 1 = reference category, Ns = not significant in bivariate analysis.

Abbreviations: AOR, adjusted odds ratio; AUD, alcohol use disorders; CI, confidence interval; COR, crude odds ratio.

^∗^Significant at *p* < 0.05. Bold denotes significant.

## Data Availability

The data supporting the findings of this study are not publicly available due to ethical and confidentiality reasons. However, they may be made available upon reasonable request.

## Nomenclature

AOR: Adjusted odds ratio
AUD: Alcohol use disorder
AUDIT: Alcohol use disorder identification test
CBOS: Community Based Organizations
CBS: Central Bureau of Statistics
CI: Confidence interval
DSM-IV: Diagnostic and Statistical Manual of Mental Disorders Fourth Edition
FCHV: Female community health volunteers
HCF: Healthcare Facilities
INGOs: International nongovernmental organization
M: Municipality
NDHS: Nepal Demographic and Health Survey
NGOs: Nongovernmental organization
NPV: Negative predictive value
OPD: Outpatient department
OR: Odds ratio
PHCs: Primary healthcare centers
PPV: Positive predictive value
RM: Rural municipality
SDG: Sustainable Development Goal
SLC: School leaving certificate
SPSS: Statistical Package for the Social Sciences
UCMS-TH: Universal College of Medical Science and Teaching Hospital
VIF: Variance Inflation Factor
WHO: World Health Organization

## Practical Application

*Implications for National Program and Health Services.* Recognizing risk factors plays a crucial role in the identification and prevention of AUD among vulnerable individuals, as well as in addressing symptoms already manifested. Directing attention toward factors linked to AUD is imperative for effective prevention and treatment strategies. This data could help policy makers to guide for different methods of intervention to reduce problems related with alcohol use. The findings of this study established the significance of formulating strategic communication programs to influence behavior. These study findings would also serve as baseline information for the future researcher.

*Areas of Further Research.* Further studies on a large scale are required to explore cultural practices, social beliefs, and value systems related to AUD among people attending primary healthcare facilities as well as in the communities, employing the qualitative design.
